# Mental health outcomes and workplace quality of life among South African pharmacists during the COVID-19 pandemic: a cross-sectional study

**DOI:** 10.1186/s40545-022-00463-7

**Published:** 2022-10-18

**Authors:** Sherishka Dhindayal, Marothi P. Letsoalo, Tanuja N. Gengiah

**Affiliations:** 1 Centre for the AIDS Programme of Research in South Africa (CAPRISA), Durban, South Africa; 2 Discipline of Pharmaceutical Sciences, School of Health Sciences, University of KwaZulu-Natal, Durban, South Africa

**Keywords:** COVID-19, Mental health, Quality-of-working life, Pharmacists, South Africa

## Abstract

**Background:**

The effect of the COVID-19 pandemic on the mental health of healthcare workers is gaining attention globally. This study assessed the quality-of-working life (QoWL) and prevalence of, and risk factors for anxiety, depression and stress among South African pharmacists.

**Methods:**

An online survey, after stratification by province, was sent to 3435 (target = 2454) randomly selected pharmacists between 14 April to 18 May 2021. Sociodemographic data were collected and mental health was assessed using the 7-item Generalized Anxiety Disorder scale, the 9-item Patient Health Questionnaire, Perceived Stress Scale and a modified Work-Related Quality-of-Life tool. Prevalence of anxiety, depression, stress and QoWL was estimated. A multivariate logistic regression analysis identified factors associated with mental health outcomes.

**Results:**

A total of 953/2454 pharmacists (38.8%) responded. Of these, 56.5% were 40 years or younger, 78.5% were female, 45.4% were White race and 44.5% were practicing in a community pharmacy setting. Pharmacists demonstrated symptoms of anxiety (*n* = 605, 66.1%), depression (*n* = 561, 62.9%), stress (*n* = 642, 73.8%) and low QoWL (*n* = 409, 51.3%). Significant risk factors (aOR; 95%CI) for anxiety, depression and stress were female gender (1.96;1.36–2.83,1.84;1.27–2.67,1.58;1.05–2.38, history of mental health conditions (2.50; 1.52–4.13, 3.68; 2.19–6.19, 3.34;1.85–6.03) and significant COVID-19 mitigation changes to pharmacy practice (2.70; 1.36–5.38, 4.23; 2.06–8.70, 3.14;1.44–6.82), respectively. Practice changes were also associated with a low QoWL (5.19; 2.40–11.8). Compared to their Black/African colleagues, Indian pharmacists were at higher risk for anxiety (1.82; 1.03–3.23) and stress symptoms (2.28; 1.21–4.32), while risk for depression was significant amongst White pharmacists (1.86; 1.05–3.32). Pharmacists living apart from family were at significant risk for anxiety (1.66; 1.15–2.41), depression (1.52; 1.06–2.18) and low QoWL (1.60; 1.10–2.34).

**Conclusions:**

COVID-19 pandemic has had a significant negative impact on the mental health of South African pharmacists. Interventions to support the psychological well-being and improve QoWL of pharmacists are needed.

## Background

The first confirmed case of COVID-19 in South Africa was reported on 05 March 2020 [1], sparking a sequence of events that ushered in an era of rapid social order change which included introducing numerous restrictive measures such as strict quarantine and staged countrywide lockdowns to limit the spread of the disease [2]. As of 07 December 2021, 21 months since the first case was reported, there has been over 3 million confirmed cases and 90 002 deaths reported in South Africa, the highest on the African continent [3, 4]. COVID-19 continues to spread in South Africa, currently experiencing its fourth wave of infections and placing the healthcare system under severe pressure [5].

Major and sustained disease outbreaks such as COVID-19 has caused physical, mental and psychological distress on society as a whole and may be attributed to increased risk for depression, anxiety, panic attacks, somatic symptoms, posttraumatic stress disorder (PTSD), psychosis, suicidal ideation and a low quality of life [6–8]. These mental health conditions could compromise work performance and increase the risk of burnout, absenteeism and resignations of health care workers (HCWs) [5, 9]. Fatigue and emotional exhaustion are likely to increase clinical errors and negatively impact patient care [5, 7]. Prioritising the physical health of society, with little attention to the mental strain that the pandemic has caused, will likely increase the risk of experiencing another ‘pandemic’ linked to the development of mental disorders [10]. Evidence from other infectious diseases outbreaks has shown that monitoring the psychological status (e.g., depression, anxiety, fatigue) of health care providers is pertinent to prevent personal exhaustion and reduced job performance [11].

The good health, both mental and physical, and the safety of HCWs, while practising their profession is important to ensure effective care of patients [12]. The risk of infection from COVID-19, lack of knowledge about the disease in the early stages of the pandemic, caring for patients with COVID-19, lack of access to personal protective equipment (PPE), wearing PPE for long hours, increased workload, long working hours, personal and professional bereavement and the fear of not having a cure available are just some of the factors that has placed HCWs under extreme stress [5, 12–15].

HCWs who have a high QoWL will be key to an effective COVID-19 response, since health care providers who are able to function optimally in their work environment will positively impact the care provided to patients [16]. It has also been demonstrated that improving QoWL reduces mental health outcomes, such as depression, anxiety and stress [16].

Pharmacists, as an important and accessible cadre of the health care workforce globally, have faced many unprecedented challenges during the COVID-19 pandemic, working tirelessly under lockdown conditions to provide continued access to medications for all ailments. Although many studies have investigated the mental health and quality of life of HCWs during the COVID-19 pandemic, the main focus has been on nurses and doctors [7–9, 17–23]. There is currently limited research conducted to understand the mental health outcomes and workplace quality of life of pharmacists during the COVID-19 pandemic. This study will provide valuable information about the mental health and workplace quality of life of pharmacists in South Africa.

## Methods

### Study design and sampling framework

This cross-sectional study was conducted using a structured online questionnaire made accessible to registered and practicing pharmacists working across nine provinces in South Africa. The survey questionnaire, hosted on SurveyMonkey^®^, was accessed via a web link sent in an invitation email with detailed information about the purpose and scope of the survey. The questionnaire consisted of 7 sections comprising a total of 83 questions and took approximately 15 min to complete. The survey was open from 14 April 2021 to 18 May 2021, representing the period after the second wave of the South African COVID-19 pandemic [24].

Stratified random sampling, based on the province, where the pharmacist was currently practicing in, was adopted to attain a representative sample. This approach ensured that the sample selected had a proportional number of participants registered to practice in each province (Table 1). The sample size was calculated using a 95% confidence interval, 5% margin of error with the assumption that the population proportion is 50% in each of the nine provinces with overall sample size of 2454. To ensure an equal selection probability within each province, the sample size was further split over the nine provinces using proportional allocation/selection probability. The sample also included an additional 40% of the required sample per province to account for potential non-respondents. The questionnaire link was then emailed to the selected 3435 pharmacists in South Africa.

**Table 1:** Sampling framework for selection of survey population

Province (geographic/practice location)	Number of registered Pharmacists in South Africa^a^	Study design			Collected data	
		Sample Size	Selection Probability	Sampling Weight	Final number of respondents	Analysis Weights
Eastern Cape	2082	361	0.173	5.767	77	27.039
Free State	482	84	0.174	5.738	26	18.538
Gauteng	4603	798	0.173	5.768	353	13.040
KwaZulu Natal	2335	405	0.173	5.765	187	12.487
Limpopo	900	156	0.173	5.769	48	18.750
Mpumalanga	732	127	0.173	5.764	29	25.241
North-West	650	113	0.174	5.752	46	14.130
Northern Cape	203	35	0.172	5.800	20	10.150
Western Cape	2163	375	0.173	5.768	167	12.952
Total	14,150	2454			953	

^a^Obtained from South African Pharmacy Council electronic database

### Participant selection

Study participants included pharmacists that are currently registered in South Africa, practicing in all pharmacy sectors. An electronic list of registered pharmacists, that included their email addresses, was obtained from the South African Pharmacy Council (SAPC) [25]. Pharmacists who were between 18 and 65 years and able to provide consent to use anonymised survey data, were eligible to participate. Pharmacists who were employed by the investigator’s primary organisation, CAPRISA (Centre for the Aids Programme of Research in South Africa) were excluded from the study to maintain confidentiality and eliminate responder bias (*n* = 8). Pharmacists whose email address and/or province were not available on the SAPC database, were excluded from the sampling framework, as they could not be invited to participate in the survey or be included in the sample stratification by province/geographic location (*n* = 165).

### Data collection

A structured questionnaire was used to collect demographic, clinical, mental health symptoms, quality-of-working life and COVID-19-related data.

The Generalized Anxiety Disorder-7 (GAD-7) scale [26] was used to measure the symptoms of anxiety. The GAD-7 tool has been used and validated in a South African setting [27, 28]. Each of these seven items is scored as 0 (never), 1 (seldom), 2 (sometimes), or 3 (often). The frequency of symptoms has been adapted to the purpose of the study. The total score for the GAD-7 ranges from 0 to 21. GAD-7 results are interpreted as follows: minimal anxiety (0–4), mild anxiety (5–9), moderate anxiety (10–14) and (15–21) indicates severe anxiety. We used a cutoff score of ≥ 10 for the presence of moderate-to-severe anxiety [26]. There is an additional, non-scored question at the end which assesses the degree to which anxiety problems has affected the patient’s level of function. Within this sample, Cronbach’s α was 0.91.

The severity of the symptoms of depression was measured using the Patient Health Questionnaire-9 (PHQ-9) [29]. PHQ-9 is a nine item tool that reflects the criteria for major depressive disorder from the Diagnostic and Statistical Manual of Mental Disorders and has been previously used in South Africa [30, 31]. Each of the nine-item tool is scored as 0 (never), 1 (seldom), 2 (sometimes), or 3 (often). The frequency of symptoms has been adapted for the purpose of the study. The total score for the PHQ-9 ranges from 0 to 27. PHQ-9 scores are categorized using a score of < 4 as minimal depression, 5–14 as moderate depression and > 15 as severe depression [29]. The cutoff score of ≥ 10 was used to categorize the presence of moderate-to-severe depression [29]. There is an additional, non-scored question at the end which assesses the degree to which depressive problems has affected the patient’s level of function. For this sample, Cronbach’s α was 0.90.

The perception of stress was assessed using the Cohen Perceived Stress Scale (PSS-10) [32]. The PSS-10 is a measure of the degree to which situations in one’s life are appraised as stressful [32] and has been previously used in South Africa [33, 34]. Likert-type responses range from 0 (never) to 4 (very often). The total score for the PSS-10 scale ranges from 0 to 40. Scores ranging from 0 to 13 are considered as low perceived stress, 14–26 as moderate perceived stress and 27–40 as high perceived stress; however, the PSS-10 does not have any diagnostic cutoff to differentiate between the stressed and not stressed individuals [32]. A cutoff score of ≥ 15 was used to categorize stressed individuals in this study and Cronbach’s α was 0.88.

To assess the quality-of-working life (QoWL) of pharmacists during COVID-19, the Work-Related Quality-of-Life (WRQoL) scale was used [35]. Several studies have used this tool to assess work-related quality of life amongst health care workers in Africa [35, 36]. It is a 23-item psychometric scale that is used to gauge the perceived quality of life of employees as measured through six psychosocial sub-factors, namely, Job and Career Satisfaction (JCS), General Well-Being (GWB), Stress at Work (SAW), Control at Work (CAW), Home–Work Interface (HWI) and Working Conditions (WCS) [35]. Item scores are derived from a 5-point Likert scale from 1 (Strongly Disagree) to 5 (Strongly Agree). A total score of 115 can be obtained. This scale has been adapted to incorporate COVID-19-related questions for the purpose of this study; therefore, in this study, the scores of nine negatively phrased items were reversed and the overall QoWL was calculated using the average of the six factors scores. Scores ranging from 23 to 71 indicated a low QoWL, 72–82 indicated an average QoWL and scores ranging from 83 to 115 indicated a high QoWL. There is no diagnostic cutoff score but, in this study, a score of ≤ 71 was used to indicate a low QoWL. The 24^th^ item on the scale measured the participant’s overall quality-of-working life. Within this sample, Cronbach’s α was 0.91.

### Statistical analysis

The data were analysed using SAS^®^ version 9.4 (SAS^®^ Institute, Cary, North Carolina) and all responses were included in the final analysis. Due to fewer frequencies, some multicategory variables were combined, such as level of qualification. The results were weighted, since the respondent/non-respondent rate was dependent on the design variable (i.e., provincial/geographic location). The non-respondents’ weights per province were, therefore, different and was calculated by taking the reciprocal of the response rate. Each weight was further divided by the sampling probability to obtain the analysis weights and estimates that are population representative and increases generalizability.

The GAD-7, PHQ-9, PSS-10 and WRQoL scales were scored as described in the relevant guidelines [26, 29, 32, 35]. The tools’ scores were presented as medians and interquartile ranges (IQRs). A chi-square test was used to test for independence between two variables and the resulting *p* values of a two-tailed test were compared to a significant level of 0.05. An *F* test, Type III method was used to assess the effect of each variable on mental health outcomes and QoWL. A design-based multiple logistic regression analysis was used to determine factors associated with moderate-to-severe anxiety (score of 10–21), moderate-to-severe depression (score of 10–27), moderate-to-high perceived stress (score of 15–40) and low QoWL (score of 0–71) taking into account the design variable. The multivariate results are reported as adjusted odds ratios and corresponding 95% confidence limits. A complete case analysis (CCA) was used for all models fitted based on the assumption that any missing data were missing at random [37].

## Results

**Fig. 1:**
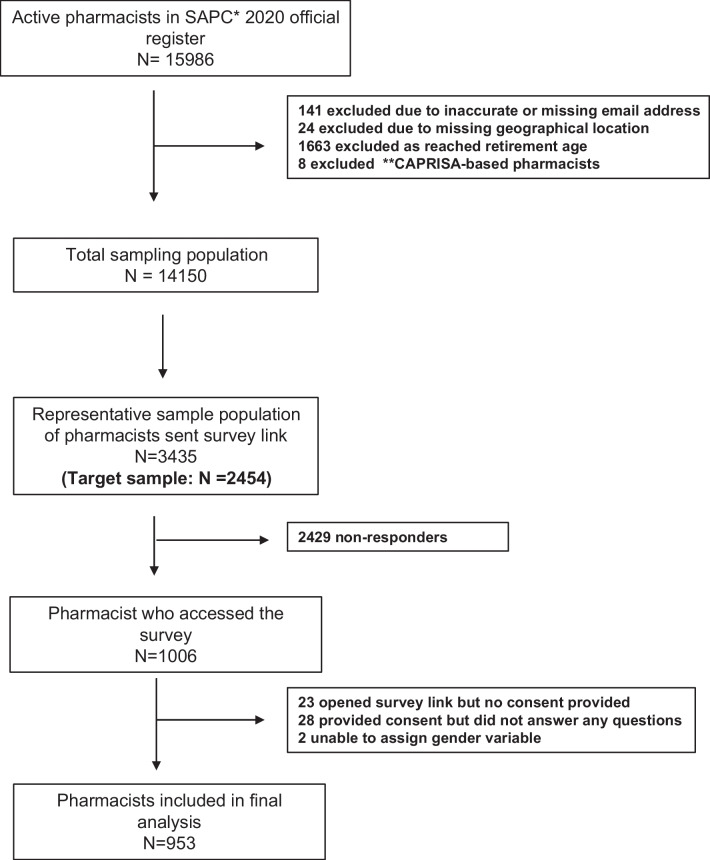
Sample selection process and final study population. *SAPC: South African Pharmacy Council, **CAPRISA: Center for the AIDS Programme of Research in South Africa, investigators organization

In this study, 3435 survey links to reach a target sample of 2454 were sent by email to pharmacists who were randomly selected to form the stratified sample described previously. A total of 953 responses were included in the final analysis. The overall response rate was 38.8% (Fig. 1).

### Pharmacists’ characteristics

The sociodemographic characteristics and clinical characteristics of pharmacists in this study (Table 2) indicate that that 56.5% are under 40 years, majority were female, 57.7% had over 10 years of pharmacist work experience and the majority practiced in the Gauteng province of South Africa. Approximately 1 in 5 of the respondents had tested positive for COVID-19 previously, 17.4% reported a pre-existing mental health condition.

**Table 2:** Sociodemographic and clinical characteristics

Variables	Categories	Participants (*n*)	% (weighted population^a^)
Demographics			
Age (years)	20–30	240	26.1
	31–40	287	30.4
	41–50	203	20.9
	51–60	171	17.5
	> 60	52	5.1
Gender	Female	748	78.5
	Male	205	21.5
Ethnicity	White	430	45.4
	Asian	246	23.6
	Black	225	25.5
	Coloured	52	5.5
Marital status	Married	583	60.7
	Never married	244	25.8
	Divorced	57	5.8
	Living together as married partners	55	6.5
	Widowed	14	1.3
Highest qualification	Bachelors	792	83.6
	Masters	139	14.1
	Doctoral	22	2.3
Professional experience (years)	0–5	190	20.4
	> 5–10	198	21.9
	> 10–20	242	25.0
	> 20	323	32.7
Current pharmacy practice sector^b^	Community/retail pharmacy	425	44.5
	Public sector hospital or clinic	229	25.7
	Pharmaceutical industry	136	13.8
	Private sector hospital or clinic	130	13.3
	Pharmaceutical wholesaler/distributor	47	4.7
	Academia	31	3.4
	Research pharmacy	24	2.3
	None of the above	61	6.1
Province (geographic/practice location)	Gauteng	353	32.5
	KwaZulu-Natal	187	16.5
	Western Cape	167	15.3
	Eastern Cape	77	14.7
	North–West	46	4.6
	Limpopo	48	6.4
	Mpumalanga	29	5.2
	Free State	26	3.4
	Northern Cape	20	1.4
Pre-existing chronic health conditions	Yes	287	29.4
	No	666	70.6
History of a mental health condition/s	Yes	164	17.4
	No	784	82.1
	*Missing*	*5*	*0.5*
Ever tested positive for COVID-19	Yes	195	20.9
	No	747	78.0
	*Missing*	*11*	*1.1*

^a^Applied to the weighted population

^b^1083 pharmacists practiced across multiple sectors during the pandemic

To better understand how the COVID-19 pandemic affected pharmacy practice, workplace access to support programs and perceived risk to COVID-19 a range of questions were posed (Table 3).

**Table 3:** The influence of the COVID-19 pandemic on pharmacy practice and workplace support

Variables	Categories	survey responses	% (weighted^a^)
Lived apart from immediate family during the pandemic	Yes	320	33.7
	No	622	65.2
	*Missing*	*11*	*1.1*
Pharmacists’ practice and/or interactions with patients affected during the COVID-19 pandemic	Not applicable to practice sector	168	17.1
	No	81	8.0
	Yes—it has been affected drastically	275	29.9
	Yes—it has been affected but only slightly	253	26.9
	*Missing*	*176*	*18.1*
How has pharmacy practice and/or interactions with patients been affected?	There was a positive impact *(I understood the situation and tried to best support my patients during the pandemic)*	324	34.5
	There was a negative impact *(I felt withdrawn and, therefore, tried to avoid interactions with my patients)*	189	20.7
	*Missing*	*15*	*44.8*
Access to stress management or stress reduction programs at workplace	Yes	224	24.8
	No	543	56.0
	*Missing*	*186*	*19.2*
Stress management or stress reduction programs/strategies available at the workplace	ICAS^b^/Telephonic support line available for staff	71	N/A
	Psychologists and Counselling/De-briefing	56	N/A
	Employee Wellness Programme	50	N/A
	Online classes and training	23	N/A
	Access to Healthcare professionals if required	4	N/A
	Extra exercise programs Home ergonomics/environment surveys and upgrades Time off at home to reflect	3	N/A
	Open door policy of immediate supervisor and management	2	N/A
	*Missing*	*15*	*N/A*
Risk perception for contracting COVID-19 at work	Low	183	19.1
	Medium	310	32.7
	High	272	28.8
	*Missing*	*188*	*19.5*
The influence of media and/or social media as a threat to mental well-being	Yes	490	52.0
	No	275	28.5
	*Missing*	*188*	*19.5*

^a^Applied to the weighted population

^b^ICAS: Independent Counselling and Advisory Services

### The emotional impact of the COVID-19 pandemic

**Fig. 2:**
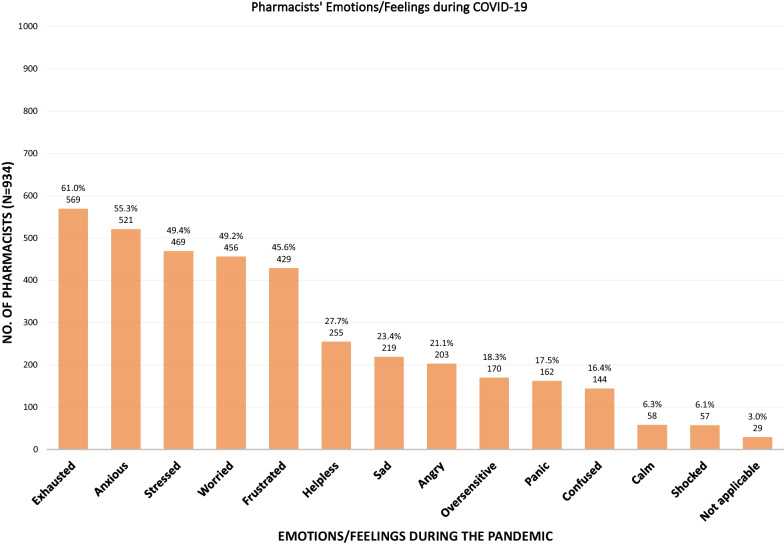
Emotions and feelings experienced during COVID-19 pandemic

Pharmacists were asked to express emotions and feelings that they experienced more than what was normal for them during the COVID-19 pandemic. These are categorised in Fig. 2.

From the structured and standardised scales previously mentioned, the prevalence of mental health outcomes (anxiety, depression, and stress) and workplace quality of life was scored and the data are presented in Table 4.

**Table 4:** The median score and prevalence of mental health outcomes

Scale	Mental health outcome	Median score [IQR]	Number of pharmacists (total respondents)	Prevalence (%)* [95% CI]
GAD-7	Generalized Anxiety	12 [8–16]	605 (919)	66.1 [62.85,69.26]
PHQ-9	Depression	12 [6–17]	561 (898)	62.9 [59.58,66.17]
PSS-10	Perceived Stress	20 [14–24]	642 (868)	73.8 [70.68,76.82]
WRQoL	Low QoWL	71 [61–83]	409 (801)	51.3 [47.67,54.93]

^*^applied to the weighted population

In addition, of the 919 respondents to the GAD-7 questionnaire, 647 (70.4%) reported that these anxiety symptoms have made it difficult for them to do their work, take care of things at home or get along with other people. Furthermore, of the 898 respondents to the PHQ-9 questionnaire, 525 (58.5%) reported that these depression symptoms have placed difficulty on their daily lives, their work and getting along with other people. In addition, of the 801 respondents to the WRQoL scale, 223 (27.6%) pharmacists were not satisfied with their overall quality-of-working life.

Results of the multivariate logistic regression used to determine potential risk factors for anxiety, depression, stress and a low QoWL are depicted in Tables 5 and 6. The results show that significant risk factors for poor mental health outcomes were female gender, living apart from family during the COVID-19 pandemic and a pre-existing poor mental health condition. Furthermore, pharmacists who indicated that their practice conditions and/or patient interactions have been drastically affected during the pandemic were twice as likely to experience anxiety, four times more likely to have depressive symptoms, three times likely to have stress and five times more likely to have low QoWL.

**Table 5:** A Design-based logistic regression Type III test for the effect of covariates^a^

Variable	GAD-7 anxiety symptoms		PHQ-9 depression symptoms		PSS-10 stress symptoms		WRQoL (low QoWL)
	*p* values						
Gender	**0.0003**	**0.0014**		**0.0279**		0.1790	
Age (years)	0.1904	0.6107		0.2718		0.3701	
Ethnicity	0.0730	**0.0488**		**0.0081**		0.5818	
Professional Experience (years)	0.5322	0.5828		0.9491		0.1949	
Qualification	0.3351	0.4935		0.9844		0.5753	
Marital Status	0.7754	0.5493		0.2492		0.0859	
History of mental health condition	**0.0003**	** < 0.0001**		**0.0001**		0.6333	
Pre-existing Chronic conditions	0.3817	0.6904		0.5792		0.4719	
Lived apart from family	**0.0072**	**0.0228**		0.0522		**0.0149**	
Pharmacists’ Practice affected during the pandemic	**0.0029**	** < 0.0001**		**0.0054**		** < 0.0001**	
Tested Positive for COVID-19	0.9171	0.8426		0.4592		0.2545	

^a^Applied to the weighted population, all p values < 0.05 are indicated in bold

**Table 6:** Multivariate logistic regression of potential risk factors for mental health outcomes in South African pharmacists^a^

Variable	*Categories (Reference)*	GAD-7 anxiety symptoms (*n* = 919) (GAD-7 ≥ 10)	PHQ-9 depression symptoms (*n* = 898) (PHQ-9 ≥ 10)	PSS-10 stress symptoms (*n* = 869) (PSS-10 ≥ 15)	WRQoL (*n* = 801) (WRQoL ≤ 71)
		aOR [CI]_95%_	aOR [CI]_95%_	aOR [CI]_95%_	aOR [CI]_95%_
Demographics					
Gender	Female (*Mal*e)	*1.961 [1.358, 2.832]	*1.837 [1.265, 2.667]	*1.580 [1.051, 2.375]	1.325 [0.879, 1.999]
Age (years)	20–30 *(*> *60)*	3.467 [0.937, 12.82]	2.118 [0.590, 7.598]	2.553 [0.646, 10.09]	1.882 [0.453, 7.810]
	31–40 *(*> *60)*	2.218 [0.724, 6.798]	1.634 [0.531, 5.027]	2.339 [0.708, 7.722]	2.370 [0.694, 8.096]
	41–50 *(*> *60)*	1.713 [0.685, 4.284]	1.677 [0.671, 4.193]	1.235 [0.484, 3.151]	1.299 [0.478, 3.533]
	51–60 *(*> *60)*	1.887 [0.792, 4.497]	1.522 [0.646, 3.587]	0.963 [0.397, 2.335]	1.190 [0.460, 3.077]
Ethnicity	Asian (*Black*)	*1.822 [1.028, 3.231]	1.410 [0.812, 2.448]	*2.284 [1.208, 4.320]	1.263 [0.695, 2.296]
	Coloured (*Black*)	1.726 [0.635, 4.690]	2.190 [0.885, 5.420]	*3.452 [1.004, 11.87]	0.874 [0.335, 2.279]
	White (*Black*)	1.250 [0.717, 2.181]	*1.862 [1.046, 3.315]	1.693 [0.905, 3.167]	0.969 [0.538, 1.745]
Professional experience (years)	> 5–10 *(0–5)*	1.532 [0.747, 3.142]	0.914 [0.459, 1.818]	1.136 [0.503, 2.569]	1.286 [0.612, 2.704]
	> 10–20 *(0–5)*	1.237 [0.513, 2.986]	0.742 [0.322, 1.713]	1.016 [0.382, 2.703]	0.650 [0.262, 1.612]
	> 20 *(0–5)*	1.122 [0.379, 3.322]	0.540 [0.185, 1.571]	0.893 [0.278, 2.865]	0.727 [0.232, 2.279]
Qualification	Doctoral (*Bachelors*)	0.458 [0.138, 1.520]	0.519 [0.134, 2.002]	1.101 [0.250, 4.844]	1.122 [0.328, 3.838]
	Masters (*Bachelors*)	0.912 [0.545, 1.527]	0.880 [0.527, 1.469]	0.977 [0.556, 1.719]	0.789 [0.466, 1.336]
Marital status	Never married *(Married)*	1.252 [0.712, 2.203]	1.015 [0.590, 1.745]	1.173 [0.605, 2.276]	1.785 [0.996, 3.198]
	Living together *(Married)*	1.132 [0.466, 2.749]	1.095 [0.450, 2.664]	0.985 [0.332, 2.922]	1.813 [0.731, 4.494]
	Divorced *(Married)*	0.798 [0.333, 1.912]	0.614 [0.261, 1.443]	0.540 [0.211, 1.383]	1.286 [0.480, 3.450]
	Widowed *(Married)*	1.363 [0.284, 6.546]	1.700 [0.348, 8.302]	2.976 [0.422, 21.02]	1.865 [0.357, 9.756]
Clinical characteristics					
History of mental health condition	Yes *(No)*	*2.504 [1.519, 4.126]	*3.682 [2.192, 6.185]	*3.337 [1.847, 6.027]	1.113 [0.717, 1.728]
Pre-existing chronic conditions	Yes *(No)*	1.183 [0.811, 1.725]	1.081 [0.736, 1.590]	0.891 [0.592, 1.341]	1.155 [0.780, 1.709]
Tested positive for COVID-19	Yes *(No)*	0.979 [0.651, 1.471]	1.042 [0.691, 1.572]	1.184 [0.757, 1.853]	1.288 [0.833, 1.990]
Influence of COVID-19 pandemic					
Lived apart from family	Yes *(No)*	*1.664 [1.148, 2.411]	*1.518 [1.060, 2.175]	1.531 [0.996, 2.353]	*1.603 [1.097, 2.344]
Pharmacists’ Practice affected during the pandemic	Yes—drastically *(No)*	*2.701 [1.356, 5.381]	*4.234 [2.060, 8.700]	*3.135 [1.442, 6.816]	*5.185 [2.404, 11.18]
	Yes—slightly *(No)*	1.401 [0.716, 2.742]	1.939 [0.969, 3.880]	1.697 [0.803, 3.585]	*2.793 [1.301, 5.995]
	Not applicable *(No)*	1.378 [0.663, 2.863]	*2.199 [1.027, 4.710]	1.649 [0.739, 3.679]	1.517 [0.668, 3.444]
	*Missing (No)*	1.783 [0.832, 3.818]	1.691 [0.764, 3.742]	1.674 [0.640, 4.375]	1.330 [0.314, 5.638]

^a^Applied to the weighted population

*aOR, CI and *p* < 0.05, aOR: adjusted odds ratio, CI_95%:_ confidence intervals at 95%

## Discussion

This cross-sectional study of 953 pharmacists revealed an unexpectedly high prevalence of mental health symptoms after the second COVID-19 wave in South Africa. Overall, the prevalence of anxiety, depression, stress and a low QoWL was 66.1%, 62.9%, 73.8% and 51.3%, respectively. Female gender, living apart from family and pre-existing mental health condition/s (reported in 17.4%) were significant risk factors for mental health outcomes. In addition, pharmacists whose practice conditions and/or patient interactions have been drastically affected during the pandemic were twice as likely to experience anxiety, four times more likely to have depressive symptoms, were at three times higher risk for stress and five times more likely to have low QoWL.

At the time of the survey, 1 in 5 of the respondents had a previous COVID-19 positive test. Furthermore, 29.4% of pharmacists had a pre-existing chronic condition, potentially increasing their risk for severe COVID-19 complications had they contracted the virus. When asked to express their emotions and feelings, the majority (> 50%) of pharmacists reported being exhausted, anxious, stressed and worried more than what they would describe as ‘normal’ for themselves. Despite these challenges, only 24.8% of the respondents reported access to stress management or stress reduction programs at their workplace and more than a third of the pharmacists lived apart from immediate family indicating a less than ideal support system in the home. The race/ethnicity of the pharmacist appeared to predict risk for mental health conditions, since compared to their Black colleagues, Asian pharmacists were at higher risk for anxiety and stress symptoms, while risk for depression was significant amongst White pharmacists.

In comparison with other studies evaluating mental health outcomes in HCWs during the first wave of infections of the COVID-19 pandemic, the prevalence of both anxiety and depression was lower in these studies, ranging from 21.1% to 51.4% for anxiety and 22.8% to 55.2% for depression, albeit the focus was primarily on doctors and nurses [20, 38–41]. In a Chinese study of frontline HCWs, the prevalence of anxiety, depression and perceived stress was 20.7% (GAD-7 ≥ 5), 45.6% (PHQ-9 ≥ 5) and 60.8% (PSS-10 ≥ 15), respectively [42]. Despite the severe COVID-19 pandemic in Italy, Rossi et al. reported a prevalence of 20.55% and 18.05% (GAD-7 ≥ 15) for anxiety and 28.35% and 19.98% (PHQ-9 ≥ 15) for depression for front-line and second-line HCWs, respectively, when comparing to general population of 21.25% for anxiety and 28.12% for depression, respectively [43]. Lai et al. and Zhang et al., both studies of Chinese HCWs, reported similar prevalence rates of 44.6% (GAD-7 ≥ 7) and 44.7% (GAD-7 ≥ 5) for anxiety and 50.4% (PHQ-9 ≥ 10) and 50.7% (PHQ-9 ≥ 5) for depression, respectively [44, 45].

Other settings with a high prevalence of anxiety, 60.2% (GAD-7 ≥ 5) and depression of 77.6% (PHQ-9 ≥ 10) was in Turkey, nurses and physicians in India had a prevalence of 50% for anxiety (GAD-7 ≥ 5), 47% for depression (PHQ-9 ≥ 5) and 45% for a low QoL (QoL-1 < 4) during the COVID-19 pandemic, while those working in Belgium had an overall prevalence of 52.2%, 53.3% and 40.3% for anxiety, depression and stress, respectively, using the DASS-21 scale [8, 21, 23]. Contrary to this study, HCWs in Nepal and Turkey reported a lower prevalence of pre-existing mental health disorders of 4.6% and 13.1%, respectively, during the COVID-19 pandemic [15, 21]. Furthermore, during the COVID-19 pandemic, these HCWs in Turkey reported a lower prevalence of 15.2% for a pre-existing chronic condition which increased their risk of COVID-19 [21].

The high prevalence of mental health outcomes observed in this cohort of South African pharmacists could be due to the restrictive lockdown measures imposed during the second wave of the pandemic which reduced social interactions that usually occurred during the summer vacation and reduced accessibility to formal psychological support. In addition, significant risk factors for mental health outcomes reported in previous studies were female gender [21, 22, 44, 46], having a history of a mental health condition [9, 15, 21] and isolating themselves away from home to protect people that they lived with [47], consistent with the results from this study. In contrast, other significant risk factors for combined depression and anxiety were marital status (being single) [8], those who tested positive for COVID-19 [21, 47] and younger HCWs (aged < 40 years) [46].

In comparison with other studies evaluating quality-of-working life, and contrary to a study conducted in Ethiopia, where only 32.7% of pharmacy professionals were satisfied with their jobs and overall quality-of-working life during the pandemic [48], this study found that 72.4% pharmacists were satisfied with their overall quality-of-working life.

Reports from previous pandemics such as severe acute respiratory syndrome (SARS), the Middle East Respiratory Syndrome (MERS), the influenza A/H1N1 (swine flu) infection and the Ebola virus further provides an insight into the huge psychological impact on HCWs and the detrimental effect on their mental health, since a large proportion of HCWs experienced anxiety, were emotionally affected and traumatized and continued to have elevated levels of psychological distress even after 1 year after the epidemic [49–52]. These studies have highlighted the need to ensure that HCWs are safe-guarded and strategies are implemented timeously to preserve their mental health during the current COVID-19 pandemic.

The impact of the COVID-19 pandemic on emotions expressed in this study was similar to other reports: in Saudi Arabia 62.2% of the HCW respondents reported negative emotions, such as fear, uncertainty, exhaustion and hopelessness [41], and Al Sulais et al. reported negative emotions, such as worry, isolation and fear experienced by nearly two-thirds of physicians [53].

In this study, compared to their Black colleagues, Asian pharmacists were at higher risk for anxiety and stress symptoms. Previous studies found that language and cultural barriers, the assertion that psychotherapy is ‘un-African’ and the fear of stigma has resulted in mental health outcomes being unrecognized and untreated in the Black and Asian communities in South Africa [54–56] and in turn may possibly be underreported. In the American context, when compared to White communities, people of colour (African Americans) had less access to mental health services, are less likely to receive treatment and are more likely to receive poor quality care when treatment was sought [57].

While prevalence of mental health outcomes is also largely not investigated and under-reported particularly amongst Black South Africans in the general population [54], it was unclear exactly why Asian pharmacists had a higher level of anxiety in comparison. In the current study Asian pharmacists were relatively young (40 years and younger) and practiced in a community/retail sector. These factors could have contributed, where being a ‘young’, front-line healthcare worker during an unprecedented pandemic could have contributed to their higher risk for anxiety, not just for their own well-being but for that of elderly family. This theory is supported in the context of the South African COVID-19 pandemic, where there were several reports of an increased risk of death from COVID-19 amongst hospitalised Asian population [58–61], possibly further contributing to their higher risk for anxiety and stress.

On the other hand, the risk for depression was significant amongst reports from White pharmacists. A large proportion of White pharmacists in this sample reported working in a community/retail (direct patient contact) pharmacy setting and the majority practiced in the Gauteng province which at the time of the study recorded the highest number of confirmed positive cases in South Africa [4] and both these factors could have contributed to increased risk for depression. Furthermore, compared to South African Blacks, where several myths and misconceptions about depression exist, White people in general, may be more likely to recognize the symptoms of depression, seek and receive medical treatment [62].

Most of the mental health outcome studies conducted in various parts of the world were conducted in 2020, during the first wave and at the peak of the COVID-19 pandemic in these respective countries and likely prior to the vaccine roll out. The use of different tools or classifications, if the same tools were utilised, to evaluate mental health outcomes may lead to reports with differing prevalence estimates. However, all studies seem to align with the premise that globally there is a significant strain on HCWs’ mental health during the COVID-19 pandemic.

It is recommended that mental health awareness, especially during pandemics, is routinely carried out to educate pharmacists about the availability of mental health care programs and services, especially at their workplace and ones that are available online and can be easily accessed [10]. Stress management or stress reduction programs and employee wellness programs, should be accessible in the workplace for pharmacists during times of crises. In addition, more attention should be paid to the mental health of vulnerable groups, such as female pharmacists, those with a history of mental health conditions and those who isolated themselves to protect the individuals that they lived with as they had a higher risk of developing anxiety, depression, high perceived stress and low quality-of-working life.

Interventions such as cognitive behavioral therapy and/or motivational interviewing have been found to be useful strategies in the management of mental health outcomes in HCWs [10] and could, therefore, be incorporated in workplace policies to assist pharmacists during a pandemic. Other effective strategies that have been found to promote better employee well-being is enhancing smart working, promoting secure protocols, trainings, increasing security and safety equipment and improving job/leadership support [10] and should be adopted in the pharmacy profession.

Since it has been established that the work environment, work organization and work-related behaviors are factors that could influence the mental health of employees, creating a safe work environment, such as a well-ventilated, sanitized, and prevention conscious workplace will result in employees being less stressed and troubled [63]. Other organizational and work-related interventions, such as improvements of workplace infrastructures, ensuring there is a regular supply of PPE available especially at the workplace and implementing resilience training programs for all employees, can also be adopted to mitigate the mental strain experienced during the pandemic [10].

Future longitudinal studies need to be carried out to follow-up on pharmacists’ mental health symptoms and develop evidence-based interventions.

### Study limitations

The study has several limitations. First, the cross-sectional design provides no information about the evolution of mental health outcomes over time, an inherent limitation when making causal inferences. Second, the investigators were reliant on the South African Pharmacy Council records for the current validity of the pharmacist’s email addresses and geographic location. It is, therefore, impossible to estimate the true participation rate and hence the representativeness of the sample. Third, those who accessed the online survey are self-selected and self-reported information was used to estimate the scores for the tools used; therefore, the outcomes may not be aligned with physician-led psychiatric assessments. The low survey response rate (38.8%) and high prevalence of pharmacists reporting a pre-existing mental health condition (17.4%), limit the generalizability of study findings. In addition, the WRQoL scale was modified to incorporate questions relating to the COVID-19 pandemic, which could affect the validity of the scale. Finally, the potential for bias exists if the true prevalence and severity of mental health outcomes in responders differs from non-responders.

Despite the several limitations, this is the first cross-sectional study conducted to evaluate the prevalence of anxiety, depression, stress, quality-of-working life and related factors in South African pharmacists and offers valuable insight into the impact of the COVID-19 pandemic on the mental health of this group of HCWs.

## Conclusions

The COVID-19 pandemic has had a significant negative impact on the mental health and quality working life of South African pharmacists. In the future, well-designed interventional studies that support the psychological well-being and improve quality-of-working life of pharmacists are needed.

## Data Availability

All data generated or analysed during this study are included in this published article.

## Abbreviations

BREC: Biomedical Research Ethics Committee
CAPRISA: Centre for the Aids Programme of Research in South Africa
CAW: Control at work
COVID-19: Coronavirus disease 2019
DASS: Depression Anxiety Stress Scale
GAD-7: Generalized Anxiety Disorder-7
GWB: General Well-being
HCW: Health care worker
HWI: Home–Work Interface
ICAS: Independent Counselling and Advisory Services
IQR: Interquartile range
JCS: Job and Career Satisfaction
MERS: Middle East Respiratory Syndrome
PHQ-9: Patient Health Questionnaire-9
PPE: Personal protective equipment
PSS: Perceived Stress Scale
PTSD: Posttraumatic stress disorder
QoWL: Quality-of-working life
SAPC: South African Pharmacy Council
SARS: Severe Acute Respiratory Syndrome
SAS: Zung Self-Rating Anxiety Scale
SAS^®^: Statistical Analysis System
SAW: Stress at work
WCS: Working Conditions
WRQoL: Work-related quality of life

## References

[1] National Institute for Communicable Diseases. First case of COVID-19 announced—an update [Internet]. 2020 (accessed 2021 Jan 13). Available from: https://www.nicd.ac.za/first-case-of-covid-19-announced-an-update/.

[2] SAnew.gov. President Ramaphosa announces a nationwide lockdown [Internet]. 2020 (accessed 2021 Feb 1). Available from: https://www.sanews.gov.za/south-africa/president-ramaphosa-announces-nationwide-lockdown.

[3] Roldan de Jong T. Perceptions of COVID-19 vaccines in South Africa, Rapid Review. Brighton: Social Science in Humanitarian Action (SSHAP). 2021.

[4] South African Department of Health. Update on Covid-19 (Tuesday 07 December 2021) [Internet]. 2021 (accessed 2021 Dec 8). Available from: https://sacoronavirus.co.za/2021/12/07/update-on-covid-19-tuesday-07-december-2021/.

[5] Robertson LJ, Maposa I, Somaroo H, Johnson O (2020). Mental health of healthcare workers during the COVID-19 outbreak: a rapid scoping review to inform provincial guidelines in South Africa. South African Med J..

[6] Müller N. Infectious Diseases and Mental Health. In: Key Issues in Mental Health. 2014; p. 99–113. Available from: https://www.karger.com/Article/FullText/365542.

[7] Stojanov J, Malobabic M, Stanojevic G, Stevic M, Milosevic V, Stojanov A. Quality of sleep and health-related quality of life among health care professionals treating patients with coronavirus disease-19. Int J Soc Psychiatry. 2020;002076402094280. 10.1177/0020764020942800. 10.1177/0020764020942800 PMC7369398 32674637

[8] Suryavanshi N, Kadam A, Dhumal G, Nimkar S, Mave V, Gupta A (2020). Mental health and quality of life among healthcare professionals during the COVID-19 pandemic in India. Brain Behav.

[9] Young KP, Kolcz DL, O’Sullivan DM, Ferrand J, Fried J, Robinson K (2021). Health Care Workers’ Mental Health and Quality of Life During COVID-19: results from a mid-pandemic, National Survey. Psychiatr Serv.

[10] Giorgi G, Lecca LI, Alessio F, Finstad GL, Bondanini G, Lulli LG (2020). COVID-19-related mental health effects in the workplace: a narrative review. Int J Environ Res Public Health.

[11] Imai H, Matsuishi K, Ito A, Mouri K, Kitamura N, Akimoto K (2010). Factors associated with motivation and hesitation to work among health professionals during a public crisis: a cross sectional study of hospital workers in Japan during the pandemic (H1N1) 2009. BMC Public Health.

[12] Dehkordi AH, Gholamzad S, Myrfendereski S, Dehkordi AH. The effect of COVID-19 on anxiety, quality of work life and fatigue of health care providers in health care centers. Res Sq. 2020;1–14.

[13] Vizheh M, Qorbani M, Arzaghi SM, Muhidin S, Javanmard Z, Esmaeili M (2020). The mental health of healthcare workers in the COVID-19 pandemic: a systematic review. J Diabetes Metab Disord.

[14] Walton M, Murray E, Christian MD (2020). Mental health care for medical staff and affiliated healthcare workers during the COVID-19 pandemic. Eur Hear J Acute Cardiovasc Care.

[15] Khanal P, Devkota N, Dahal M, Paudel K, Joshi D (2020). Mental health impacts among health workers during COVID-19 in a low resource setting: a cross-sectional survey from Nepal. Global Health.

[16] Bakhshi E, Moradi A, Naderi M, Kalantari R (2018). Associations of the quality of work life and depression, anxiety, and stress in the employees of healthcare systems. J Patient Saf Qual Improv.

[17] Kang L, Li Y, Hu S, Chen M, Yang C, Yang BX (2020). The mental health of medical workers in Wuhan, China dealing with the 2019 novel coronavirus. Lancet Psychiatry.

[18] Li Q, Chen J, Xu G, Zhao J, Yu X, Wang S (2020). The psychological health status of healthcare workers during the COVID-19 outbreak: a cross-sectional survey study in Guangdong, China. Front Public Health.

[19] Tan BYQ, Chew NWS, Lee GKH, Jing M, Goh Y, Yeo LLL (2020). Psychological impact of the COVID-19 pandemic on health care workers in Singapore. Ann Intern Med.

[20] Al Ammari M, Sultana K, Thomas A, Al Swaidan L, Al Harthi N. Mental health outcomes amongst health care workers during COVID 19 pandemic in Saudi Arabia. Front Psychiatry. 2021;11. 10.3389/fpsyt.2020.619540 PMC7840896 33519559

[21] Şahin MK, Aker S, Şahin G, Karabekiroğlu A (2020). Prevalence of depression, anxiety, distress and insomnia and related factors in healthcare workers during COVID-19 pandemic in Turkey. J Community Health.

[22] Buselli R, Corsi M, Baldanzi S, Chiumiento M, Del LE, Dell’oste V (2020). Professional quality of life and mental health outcomes among health care workers exposed to SARS-CoV-2 (COVID-19). Int J Environ Res Public Health.

[23] Tiete J, Guatteri M, Lachaux A, Matossian A, Hougardy JM, Loas G (2021). Mental health outcomes in healthcare workers in COVID-19 and non-COVID-19 care units: a cross-sectional survey in Belgium. Front Psychol.

[24] National Institute for Communicable Diseases. COVID-19 Special Public Health Surveillance Bulletin [Internet]. 2021. Available from: https://www.nicd.ac.za/wp-content/uploads/2021/03/COVID-19-Special-Public-Health-Surveillance-Bulletin-9-12-March-2021.pdf.

[25] South African Pharmacy Council. South African Pharmacy Council - Order form for Pharmacy and Pharmacist List [Internet]. 2021 (accessed 2021 Mar 23). Available from: www.sapc.za.org.

[26] Spitzer RL, Kroenke K, Williams JBW, Löwe B (2006). A brief measure for assessing generalized anxiety disorder. Arch Intern Med.

[27] Adjorlolo S. Generalised anxiety disorder in adolescents in Ghana: examination of the psychometric properties of the generalised anxiety disorder-7 scale. African J Psychol Assess. 2019;1. Available from: https://ajopa.org/index.php/ajopa/article/view/10

[28] Haas AD, Technau K, Pahad S, Braithwaite K, Madzivhandila M, Sorour G, et al. Mental health, substance use and viral suppression in adolescents receiving ART at a paediatric HIV clinic in South Africa. J Int AIDS Soc. 2020;23(12). 10.1002/jia2.25644 10.1002/jia2.25644 PMC7720277 33283916

[29] Kroenke K, Spitzer RL, Williams JBW (2001). The PHQ-9. J Gen Intern Med.

[30] Cholera R, Gaynes BN, Pence BW, Bassett J, Qangule N, Macphail C (2014). Validity of the patient health questionnaire-9 to screen for depression in a high-HIV burden primary healthcare clinic in Johannesburg, South Africa. J Affect Disord.

[31] Bhana A, Rathod SD, Selohilwe O, Kathree T, Petersen I (2015). The validity of the Patient Health Questionnaire for screening depression in chronic care patients in primary health care in South Africa. BMC Psychiatry.

[32] Cohen S, Kamarck T, Mermelstein R (1983). A global measure of perceived stress. J Health Soc Behav.

[33] Hamad R, Fernald LCH, Karlan DS, Zinman J (2008). Social and economic correlates of depressive symptoms and perceived stress in South African adults. J Epidemiol Commun Health..

[34] Makhubela M (2020). Assessing psychological stress in South African university students: measurement validity of the perceived stress scale (PSS-10) in diverse populations. Curr Psychol.

[35] Van Laar D, Edwards JA, Easton S (2007). The work-related quality of Life scale for healthcare workers. J Adv Nurs.

[36] Opollo J, Gray J, Spies L (2014). Work-related quality of life of Ugandan healthcare workers. Int Nurs Rev.

[37] Hughes RA, Heron J, Sterne JAC, Tilling K (2019). Accounting for missing data in statistical analyses: multiple imputation is not always the answer. Int J Epidemiol.

[38] Li W, Yang Y, Ng CH, Zhang L, Zhang Q, Cheung T, et al. Global imperative to combat stigma associated with the coronavirus disease 2019 pandemic. Psychol Med. 2020;1–2. Available from: https://www.cambridge.org/core/product/identifier/S0033291720001993/type/journal_article. 10.1017/S0033291720001993 PMC7261961 32450925

[39] Pappa S, Ntella V, Giannakas T, Giannakoulis VG, Papoutsi E, Katsaounou P (2020). Prevalence of depression, anxiety, and insomnia among healthcare workers during the COVID-19 pandemic: a systematic review and meta-analysis. Brain Behav Immun.

[40] Chung JPY, Yeung WS (2020). Staff mental health self-assessment during the COVID-19 outbreak. East Asian Arch Psychiatry.

[41] AlAteeq DA, Aljhani S, Althiyabi I, Majzoub S (2020). Mental health among healthcare providers during coronavirus disease (COVID-19) outbreak in Saudi Arabia. J Infect Public Health.

[42] Tian T, Meng F, Pan W, Zhang S, Cheung T, Ng CH, et al. Mental health burden of frontline health professionals treating imported patients with COVID-19 in China during the pandemic. Psychol Med. 2020;1–2. Available from: https://www.cambridge.org/core/product/identifier/S0033291720002093/type/journal_article. 10.1017/S0033291720002093 PMC7324657 32468966

[43] Rossi R, Socci V, Pacitti F, Mensi S, Di Marco A, Siracusano A, et al. Mental Health Outcomes Among Healthcare Workers and the General Population During the COVID-19 in Italy. Front Psychol. 2020;11. 10.3389/fpsyg.2020.608986/full. 10.3389/fpsyg.2020.608986 PMC7753010 33363500

[44] Lai J, Ma S, Wang Y, Cai Z, Hu J, Wei N (2020). Factors associated with mental health outcomes among health care workers exposed to coronavirus disease 2019. JAMA Netw Open.

[45] Zhang C, Yang L, Liu S, Ma S, Wang Y, Cai Z, et al. Survey of insomnia and related social psychological factors among medical staff involved in the 2019 novel coronavirus disease outbreak. Front Psychiatry. 2020;11. 10.3389/fpsyt.2020.00306/full. 10.3389/fpsyt.2020.00306 PMC7171048 32346373

[46] Conti C, Fontanesi L, Lanzara R, Rosa I, Porcelli P (2020). Fragile heroes. The psychological impact of the COVID-19 pandemic on health-care workers in Italy. PLoS ONE.

[47] Firew T, Sano ED, Lee JW, Flores S, Lang K, Salman K (2020). Protecting the front line: a cross-sectional survey analysis of the occupational factors contributing to healthcare workers’ infection and psychological distress during the COVID-19 pandemic in the USA. BMJ Open.

[48] Ayele Y, Hawulte B, Feto T, Basker GV, Bacha YD (2020). Job satisfaction among pharmacy professionals working in public hospitals and its associated factors, eastern Ethiopia. J Pharm Policy Pract.

[49] Goulia P, Mantas C, Dimitroula D, Mantis D, Hyphantis T (2010). General hospital staff worries, perceived sufficiency of information and associated psychological distress during the A/H1N1 influenza pandemic. BMC Infect Dis.

[50] Chan AOM, Chan YH (2004). Psychological impact of the 2003 severe acute respiratory syndrome outbreak on health care workers in a medium size regional general hospital in Singapore. Occup Med (Chic Ill).

[51] Lee AM, Wong JGWS, Mcalonan GM, Cheung V, Cheung C, Sham PC (2007). Stress and psychological distress among SARS survivors 1 year after the outbreak. Can J Psychiatry.

[52] Lehmann M, Bruenahl CA, Löwe B, Addo MM, Schmiedel S, Lohse AW (2015). Ebola and psychological stress of health care professionals. Emerg Infect Dis.

[53] Al Sulais E, Mosli M, AlAmeel T (2020). The psychological impact of COVID-19 pandemic on physicians in Saudi Arabia: a cross-sectional study. Saudi J Gastroenterol.

[54] The South African Depression and Anxiety Group. DEPRESSION IN BLACK SOUTH AFRICANS [Internet]. 2021 (accessed 2021 Aug 26). Available from: https://www.sadag.org/index.php?option=com_content&view=article&id=645:depression-in-black-south-africans&catid=84&Itemid=120.

[55] Knight ZG (2013). Black client, white therapist: Working with race in psychoanalytic psychotherapy in South Africa. Int J Psychoanal.

[56] Padayachee P, Laher S (2014). South African hindu psychologists’ perceptions of mental illness. J Relig Health.

[57] McGuire TG, Miranda J (2008). New evidence regarding racial and ethnic disparities in mental health: policy implications. Health Aff.

[58] Jassat W, Brey Z, Parker S, Wadee M, Wadee S, Madhi SA (2021). A call to action: temporal trends of COVID-19 deaths in the South African Muslim community. South African Med J..

[59] Hughes GD, Mbamalu ON, Okonji CO, Puoane TR (2022). The impact of health disparities on COVID-19 outcomes: early findings from a high-income country and two middle-income countries. J Racial Ethn Heal Disparities..

[60] Jassat, W, Cohen, C, Tempia, S et al. A national cohort study of COVID-19 in-hospital mortality in South Africa: the intersection of communicable and non-communicable chronic diseases in a high HIV prevalence setting. BMJ Yale. 2020.

[61] National Institute for Communicable Diseases. COVID-19 SENTINEL HOSPITAL SURVEILLANCE UPDATE [Internet]. 2020. Available from: https://www.nicd.ac.za/wp-content/uploads/2020/09/NICD-COVID-19-Weekly-Sentinel-Hospital-Surveillnace-update-Week-38-2020-updated.pdf.

[62] MindDoc. Depression In South Africa’s Black Community. Available from: https://minddoc.de/magazin/en/depression-south-africa/.

[63] Tan W, Hao F, McIntyre RS, Jiang L, Jiang X, Zhang L (2020). Is returning to work during the COVID-19 pandemic stressful? A study on immediate mental health status and psychoneuroimmunity prevention measures of Chinese workforce. Brain Behav Immun.

